# The currency, completeness and quality of systematic reviews of acute management of moderate to severe traumatic brain injury: A comprehensive evidence map

**DOI:** 10.1371/journal.pone.0198676

**Published:** 2018-06-21

**Authors:** Anneliese Synnot, Peter Bragge, Carole Lunny, David Menon, Ornella Clavisi, Loyal Pattuwage, Victor Volovici, Stefania Mondello, Maryse C. Cnossen, Emma Donoghue, Russell L. Gruen, Andrew Maas

**Affiliations:** 1 Australian and New Zealand Intensive Care Research Centre (ANZIC-RC), School of Public Health and Preventive Medicine, Monash University, Melbourne, Victoria, Australia; 2 National Trauma Research Institute, The Alfred, Monash University, Melbourne, Victoria, Australia; 3 Cochrane Australia, School of Public Health and Preventive Medicine, Monash University, Melbourne, Victoria, Australia; 4 Cochrane Consumers and Communication, School of Psychology and Public Health, La Trobe University, Melbourne, Victoria, Australia; 5 BehaviourWorks Australia, Monash Sustainable Development Institute, Monash University, Melbourne, Victoria, Australia; 6 Division of Anaesthesia, University of Cambridge; Neurosciences Critical Care Unit, Addenbrooke’s Hospital; Queens’ College, Cambridge, United Kingdom; 7 MOVE: Muscle, Bone and Joint Health Ltd, Melbourne, Victoria, Australia; 8 Monash Centre for Occupational and Environmental Health (MonCOEH), Monash University, Melbourne, Victoria, Australia; 9 Department of Public Health, Erasmus MC University Medical Center, Rotterdam, The Netherlands; 10 Department of Neurosurgery, Erasmus MC University Medical Center, Rotterdam, The Netherlands; 11 Department of Biomedical and Dental Sciences and Morphofunctional Imaging, University of Messina, Messina, Italy; 12 Center for Medical Decision Making, Department of Public Health, Erasmus Medical Center, Rotterdam, The Netherlands; 13 Nanyang Technical University, Singapore; 14 Central Clinical School, Monash University, Melbourne, Victoria, Australia; 15 Department of Neurosurgery, Antwerp University Hospital and University of Antwerp, Edegem, Belgium; Cardiff University, UNITED KINGDOM

## Abstract

**Objective:**

To appraise the currency, completeness and quality of evidence from systematic reviews (SRs) of acute management of moderate to severe traumatic brain injury (TBI).

**Methods:**

We conducted comprehensive searches to March 2016 for published, English-language SRs and RCTs of acute management of moderate to severe TBI. Systematic reviews and RCTs were grouped under 12 broad intervention categories. For each review, we mapped the included and non-included RCTs, noting the reasons why RCTs were omitted. An SR was judged as ‘current’ when it included the most recently published RCT we found on their topic, and ‘complete’ when it included every RCT we found that met its inclusion criteria, taking account of when the review was conducted. Quality was assessed using the AMSTAR checklist (trichotomised into low, moderate and high quality).

**Findings:**

We included 85 SRs and 213 RCTs examining the effectiveness of treatments for acute management of moderate to severe TBI. The most frequently reviewed interventions were hypothermia (n = 17, 14.2%), hypertonic saline and/or mannitol (n = 9, 7.5%) and surgery (n = 8, 6.7%). Of the 80 single-intervention SRs, approximately half (n = 44, 55%) were judged as current and two-thirds (n = 52, 65.0%) as complete. When considering only the most recently published review on each intervention (n = 25), currency increased to 72.0% (n = 18). Less than half of the 85 SRs were judged as high quality (n = 38, 44.7%), and nearly 20% were low quality (n = 16, 18.8%). Only 16 (20.0%) of the single-intervention reviews (and none of the five multi-intervention reviews) were judged as current, complete and high-quality. These included reviews of red blood cell transfusion, hypothermia, management guided by intracranial pressure, pharmacological agents (various) and prehospital intubation. Over three-quarters (n = 167, 78.4%) of the 213 RCTs were included in one or more SR. Of the remainder, 17 (8.0%) RCTs post-dated or were out of scope of existing SRs, and 29 (13.6%) were on interventions that have not been assessed in SRs.

**Conclusion:**

A substantial number of SRs in acute management of moderate to severe TBI lack currency, completeness and quality. We have identified both potential evidence gaps and also substantial research waste. Novel review methods, such as Living Systematic Reviews, may ameliorate these shortcomings and enhance utility and reliability of the evidence underpinning clinical care.

## Introduction

Systematic reviews, as rigorous and replicable summaries of the existing research, have long been considered a cornerstone of evidence based medicine [1, 2]. Systematic reviews inform clinical care by underpinning clinical practice guidelines [3, 4] and guide future research by summarising what is known and highlighting what is unknown on a topic [5].

As part of growing interest in increasing value and reducing waste in research [6, 7], there have been renewed calls for well-conducted systematic reviews to underpin all proposals for new primary research [8]. Yet, making sense of what is likely to be numerous evidence syntheses on a specific topic is increasingly challenging [9]. Systematic reviews are growing exponentially: current estimates suggest that over 8,000 systematic reviews are published annually [10]. A further complication is that many systematic reviews are poorly conducted and reported [10], with unnecessary duplication of topics, and conflicting or misleading results common [9, 11].

The need for well-conducted and up-to-date evidence syntheses to inform clinical care and future research is particularly pertinent within the context of traumatic brain injury (TBI). TBI is a global health concern [12], with often devastating and ongoing physical and cognitive impairments, and substantial financial and social costs to individuals, families and communities [13]. In the area of acute management of moderate to severe TBI, approximately 200 randomised controlled trials (RCTs) have been conducted, exploring a myriad of pharmacological, surgical and other treatments [14]. To date, however, TBI trials have largely shown disappointing results, with relatively few interventions underpinned by convincing evidence to support their use [14–16]. Strategic TBI research planning is therefore critical, such that research resources can be directed to areas of need and duplication of effort is avoided.

Broad overviews of the existing and emerging evidence can facilitate such planning [5]. Previous authors have conducted overviews of a select systematic reviews of acute management of moderate to severe TBI (Cochrane Reviews only [17]; pharmacological treatments only [18]). Others have reviewed the findings, quality and reporting of RCTs in this area [14, 19, 20]. Bragge et al [21] mapped the primary and secondary research in TBI against stakeholder-prioritised research questions. To our knowledge, however, no-one has comprehensively examined systematic reviews across the entire field of acute management of moderate to severe TBI to determine their trustworthiness to inform clinical care and research.

As such, the aim of this research was to appraise the currency, completeness and quality of evidence from systematic reviews of acute management of moderate to severe TBI.

## Methods

We applied an evidence mapping approach to primary and secondary research for the acute management of moderate to severe TBI. Evidence maps describe the quantity, design and characteristics of research in broad topic areas; providing a snapshot of what it is known and where evidence is lacking [21, 22]. Given the absence of reporting checklists for evidence maps [22], we followed the applicable sections of the PRISMA checklist for reporting systematic reviews [23].

### Eligibility criteria

We included published, English-language systematic reviews and RCTs of interventions for the acute management of moderate to severe TBI across all participant age groups.

We used the PRISMA definition of a systematic review [23], applying the following minimum standards for inclusion: explicit inclusion criteria and search strategy reported, and provided a complete account of their included studies. Overviews of reviews were excluded as these are redundant within this project; narrative reviews were excluded, as were systematic reviews that did not seek to include RCTs (as stated in their inclusion criteria). Where a systematic review had been updated (for example, a Cochrane Review), we included the most recent version only.

We used the Cochrane definition of an RCT, in that participants were definitely or probably assigned prospectively to one of two (or more) groups using random allocation [24]. We excluded quasi-random RCTs (whereby the method of allocation was not truly random, such as day of the week).

Moderate to severe TBI was defined as a Glasgow Coma Scale (GCS) score ≤ 12, however we did not exclude reviews and RCTs if they only referred to moderate to severe TBI without providing a GCS-based definition. Acute management was defined as any intervention delivered in the pre-hospital or acute setting. Interventions delivered in the rehabilitation setting were excluded. Where systematic reviews included mixed populations (i.e. mild TBI or non-TBI, such as stroke) we included the review, but excluded these specific RCTs from our analysis. RCTs with mixed populations were excluded.

### Searching

Initial searches were conducted in March 2015, with update searches in March 2016. For the 2015 search, we utilised an existing neurotrauma evidence synthesis repository, the Neurotrauma EvidenceMap [25], previously managed by some members of the author team, to search for systematic reviews. Comprehensive searches of Medline, Embase, CINAHL Plus and the Cochrane Library underpin the repository, with two screeners independently deciding on included reviews [18]. To search for RCTs, we utilised an overview of RCTs by Bragge et al [14], given their comprehensive searching of Medline, All Evidence based Medicine Reviews (OVID), EMBASE and CENTRAL in March 2015. Further RCTs were identified from the included study lists of the included systematic reviews.

For the 2016 search, we searched for systematic reviews in Epistemonikos [26], an evidence synthesis repository that employs continual searches of 26 health databases. For RCTs, we searched Cochrane’s CENTRAL, a composite database of (predominantly) RCTs found in Medline, EMBASE, and hand searching of journals. For both sources, we tailored a search string with TBI keywords and MESH terms (as appropriate), searching for articles published between January 2015 to March 2016 (see S1 File).

### Screening

For the 2015 search, given two independent screeners had already determined the inclusion of systematic reviews into the Neurotrauma Evidence Map repository, one reviewer (AS) downloaded into Microsoft Word the systematic review titles grouped together under management of TBI and screened them on title and then full-text to determine eligibility. Any uncertainties were discussed and resolved with another reviewer (PB, LP). Given we used identical eligibility criteria as Bragge et al [14] (and screening for that review was conducted by PB and AS) we did not rescreen their included RCTs, instead including them all directly as included RCTs in this paper (with the exception of any that were still ongoing).

For the 2016 yield, two reviewers (two of MC, SM, VV or ED) independently screened citations for both systematic review and RCT searches on title and abstract using the online software program, Covidence. One reviewer (ED) then screened citations on full-text, which were checked by a second reviewer (AS). Any uncertainties were resolved by discussion.

### Data extraction

One reviewer (AS) extracted the following characteristics for each systematic review: (1) year of publication and year of search, (2) participants (adult or paediatric, eligibility criteria), (3) intervention(s) and comparisons (type, dose and dose regimen, if relevant), and (4) the list of included RCTs. The same reviewer extracted the following RCT characteristics: (1) year or publication, (2) participants (adult or paediatric), (3) intervention(s) and comparisons (type, dose and timing, if relevant), and (4) outcomes (if relevant to specific systematic review inclusion criteria).

### Quality assessment

We assessed the methodological quality of systematic reviews using the 11-item AMSTAR checklist [27], a valid and reliable quality assessment tool [28]. Systematic reviews retrieved from Neurotrauma EvidenceMap [25] were independently assessed by two authors (LP, OC or AS). For the more recent systematic reviews found in Epistemonikos [26], one reviewer (AS) assessed quality with the AMSTAR tool. To facilitate comparisons between systematic reviews, we grouped AMSTAR scores into the following quality categories: low (0 to 3), moderate (4 to 7) and high (8 to 11), according to the categories used in a Cochrane overview of systematic reviews [29].

### Mapping approach

The mapping process was performed by one author (AS) in Microsoft Excel (2007), and involved the following steps:

1. Systematic reviews and RCTs were grouped by topic into 12 intervention categories (and then further, into ‘like’ interventions), based on those used by Bragge et al [14] and in discussion with the clinical authors. The 12 intervention categories included, (1) Airway, ventilation and oxygenation strategies, (2) Fluid management, (3) Hypothermia, (4) Intracranial, cerebral and blood pressure management, (5) Nutrition and glucose management, (6) Pharmacological therapies not elsewhere defined, (7) Glutamate receptor antagonists, (8) Prehospital and systems of care, (9) Sedation, pain management, anaesthesia and arousal, (10) Seizure prophylaxis, (11) Corticosteroids, and (12) Surgery. Systematic reviews that included multiple interventions (‘multi-intervention’ reviews) were ‘split’, so that each intervention included in the review was considered within its appropriate intervention category.
2. Systematic reviews and RCTs within each of the 12 intervention categories were plotted against each other. This involved cataloguing which RCTs were included /not included in each of the systematic reviews. In some instances, the systematic reviews included RCTs that did not meet our inclusion criteria (e.g. non-English language, not truly random allocation, mixed population, duplicate publication referring to an already included study). In these instances those RCTs were removed from the analysis, and not counted as one of the included studies in that systematic review.
3. The non-included trials were then classified by comparing the PICO (participants, intervention, comparison, outcomes) information of each RCT with the systematic review inclusion criteria. One of three classifications was assigned to each RCT:
  - Post-date the review: The trial appeared to meet the systematic review inclusion criteria but was published during or after the year the review’s search was conducted.
  - Out of Scope: The trial did not appear to meet the systematic review inclusion criteria (irrespective of when it was published).
  - True Missing: The trial was missing from the review despite meeting the systematic review inclusion criteria and being published within the review search dates. Where it was not possible to definitively classify an RCT as ‘post-date’ or ‘out of scope’ due to lack of information reported, it was classified as ‘true missing’.

Where there was uncertainty regarding classification, it was discussed with another member of the author team (PB, OC, VV, CL or ED) until a decision was reached.

### Assessment of currency, completeness and quality

This mapping process allowed us to assess the currency, completeness, and quality of each systematic review. These terms were defined in the following ways:

- Currency: When the systematic review included the most recently published trials. A review was considered ‘current’ when it had no RCTs classified as ‘post-date the review’, and not ‘current’ when it had one or more RCTs classified as ‘post-date the review’.
- Completeness: Whether the systematic review captured all known RCTs that met its inclusion criteria, relative to when it was conducted. A review was considered ‘complete’ when it had no RCTs classified as ‘true missing’, and ‘incomplete’ when it had one or more RCT classified as ‘true missing’.
- Quality was defined as the methodological quality of the review, as measured by the AMSTAR checklist [27]. Reviews were classified as high (score of 8 to 11), moderate (score of 4 to 7) or low (score 0 to 3) quality using an approach that has previously been applied to an overview of systematic reviews [29].

### Visual presentation of currency, completeness and quality

The findings relating to currency, completeness and quality of systematic reviews were presented visually, using the bubble plot format [22]. Bubble plots use four dimensions to display information: the size and colour of the bubble, and the x- and y- axes. In our bubble plot, each bubble represents a single systematic review. To facilitate ease of display, we grouped the data together in the following ways:

- Size of the bubble: represents the number of included RCTs in the systematic review, from 0 to 5 (small), 6 to 10 (medium) and 11 or more (large).
- Colour: represents currency, with current (green) or not current (red)
- X-axis: represents systematic review quality, as low, moderate or high
- Y-axis: represents completeness, with the number of RCTs defined as true missing grouped into three categories (0, 1 to 2 and 3 or more)

## Results

### Search results

We identified 67 systematic reviews from existing resources in the March 2015 search (see S1 Fig). In the 2016 update, we screened 1,092 systematic review citations on title and abstract, obtaining 91 of these in full-text. We included 19 systematic reviews, bringing the total number of included systematic reviews to 85. A list of key systematic reviews (meaning those a reader may reasonably expect to find in the review[30]) excluded on full-text is provided in S1 Table.

For RCTs, we included 194 RCTs in March 2015 from existing resources, and screened a further 672 citations on title and abstract in March 2016. Of these, 47 were screened in full-text and 19 RCTs were included to the original yield, bringing the total number of included RCTs to 213 (see S1 Fig).

### Included systematic reviews and randomised controlled trials

We included 85 systematic reviews and 213 RCTs, examining the effectiveness of a range of interventions for the acute management of moderate to severe TBI. Eighty systematic reviews assessed a single intervention (‘single-intervention reviews’) and the remaining five reviews each assessed multiple interventions (‘multi-intervention reviews’ [19, 31, 32]). Given the five multi-intervention reviews were effectively split into 40 single-intervention reviews to facilitate the mapping process, we considered the currency and completeness of these reviews separately.

The interventions featured in the most systematic reviews included hypothermia (n = 17, 14.2%), hypertonic saline and/or mannitol (n = 9, 7.5%) and surgery (n = 8, 6.7%). Progesterone, monoaminergic agonists and nutrition (timing, delivery route and elements) were the topic of five (4.2%) systematic reviews each, with barbiturates, corticosteroids, antifibrinolytic agents, hyperventilation and hyperbaric hyperoxia each featuring in four (3.3%) systematic reviews.

Of the 85 systematic reviews, the majority (n = 56, 65.9%) included participants of any age, while approximately one third (n = 25, 29.4%) included only adults. Only four (4.7%) systematic reviews focussed solely on paediatric populations. Single-intervention reviews included a median of two RCTs (range 0 to 20 RCTs), whereas the multi-intervention reviews included a mean of 22 RCTs (range 3 to 47 RCTs).

### Currency, completeness and quality across systematic reviews

Key systematic review characteristics and quality scores, the number of included RCTs, and the number of non-included RCTs (classified by reason for non-inclusion) for each systematic review are presented in Table 1. In the table, the reasons for non-inclusion of RCTs have been shortened to PD (post-dates the systematic review), S (out of scope) and T (true missing).

**Table 1:** Systematic review characteristics and quality, with number of included and non-included RCTs.

Systematic review	Pop.	Intervention (vs comparison)	Search	RCTs	Qual.	Non-includ. RCTs		
						PD*	S^^^	T^#^
**1. Airway, ventilation and oxygenation strategies**								
**Hyperbaric hyperoxia**								
McDonough 2004^[^^33^^]^	All	Hyperbaric oxygen therapy**	2003	2	Mod	3	1	1
^§^Meyer 2010^[^^31^^]^	All	Hyperbaric oxygen therapy	1980–2008	2	Mod	3	1	1
^§^Lu 2012^[^^19^^]^	Adult	Hyperbaric oxygen therapy	2011	2	Low	1	2	2
Bennett 2012^[^^34^^]^	All	Hyperbaric oxygen therapy	2012	5	High	1	1	0
**Hyperventilation**								
^§^Roberts 1998^[^^32^^]^	All	Hyperventilation vs. normovent.	1996	1	Low	0	0	0
Roberts 1997^[^^35^^]^	All	Hyperventilation	2008	1	Mod	0	0	0
^§^Meyer 2010^[^^31^^]^	All	Hyperventilation	1980–2008	1	Mod	0	0	0
^§^Lu 2012^[^^19^^]^	Adult	Hyperventilation	2011	1	Low	0	0	0
**Management guided by brain tissue oxygen**								
Nangunoori 2012^[^^36^^]^	All	PbtO_2_-based vs ICP/CPP-based**	1993–2010	0	Low	0	5	0
Lazaridis 2014^[^^37^^]^	Adult	Monitoring (≥ 2: PbtO_2_, PRx, LPR)**	2013	4	Mod	0	1	0
**2. Fluid management**								
**Blood or blood product transfusion**								
Nishijima 2012^[^^38^^]^	Adult	Platelet transfusion	2011	0	Mod	0	3	0
*†Boutin 2015*^*[*^^*39*^^*]*^	*Adult*	*RBC transfusion*	*2015*	*2*	*High*	*0*	*1*	*0*
**3. Hypothermia**								
**Hypothermia**								
Harris 2002^[^^40^^]^	Adult	Hypothermia vs. normo.**	?2001	7	Mod	14	11	4
McIntyre 2003^[^^41^^]^	Adult	Hypothermia vs. normo.**	2002	11	High	13	11	1
Henderson 2003^[^^42^^]^	All	Hypothermia**	2002	8	Mod	21	5	2
Peterson 2008^[^^43^^]^	Adult	Hypothermia vs. SC	2007	12	Mod	10	6	8
*†Saxena 2008*^*[*^^*44*^^*]*^	*All*	*Hypothermia min*. *35*^*◦*^ *C*	*2008*	*0*	*High*	*0*	*36*	*0*
Sydenham 2009^[^^45^^]^	All	Hypothermia max. 35^◦^ C	2009	20	High	10	5	1
^§^Meyer 2010^[^^31^^]^	All	Hypothermia	1980–2008	9	Mod	12	5	10
Fox 2010^[^^46^^]^	Adult	Early hypothermia vs normo.**	?2008	11	High	5	18	2
Sadaka 2012^[^^47^^]^	Adult	Hypothermia**	2010	8	Low	7	19	2
Georgiou 2013^[^^48^^]^	All	Systemic hypothermia**	2011	17	High	5	13	1
*†*Harris 2012^[^^49^^]^	Adult	Non-invasive head cooling	2011	1	High	0	35	0
^§^Lu 2012^[^^19^^]^	Adult	Hypothermia	2011	8	Low	5	5	18
Ma 2013^[^^48^^]^	Paed.	Hypothermia vs normo.**	?2012	3	Mod	2	31	0
Crossley 2014^[^^50^^]^	Adult	Hypothermia**	2012	15	High	1	18	2
Li 2014^[^^51^^]^	Adult	Moderate hypothermia	2012	11	Mod	1	19	5
Madden 2015^[^^52^^]^	Adult	Hypothermia**	2009–2013	2	Low	1	33	0
Zhang 2015^[^^53^^]^	Paed.	Hypothermia**	2014	4	Mod	1	31	0
**4. Intracranial, Cerebral Perfusion and Blood Pressure management**								
**Hypertonic saline and/or mannitol**								
^§^Roberts 1998^[^^32^^]^	All	Mannitol vs. no mannitol	1996	1	Low	0	17	0
Banks 2008^[^^54^^]^	All	HTS	2007	4	Low	0	14	0
^§^Meyer 2010^[^^31^^]^	All	Mannitol, and/or HTS	1980–2008	10	Low	6	0	2
Wakai 2013^[^^55^^]^	All	Mannitol	2009	4	High	2	12	0
Kamel 2011^[^^56^^]^	All	Mannitol vs. HTS**	2010	1	Mod	2	14	1
^§^Lu 2012^[^^19^^]^	Adult	Mannitol, and/or HTS	2011	5	Low	3	2	8
Rickard 2014^[^^57^^]^	Adult	Mannitol vs. HTS**	?2012	3	Mod	0	14	1
Lourens 2014^[^^58^^]^	All	HTS vs. saline/Lactated Ringers**	2011	3	High	1	13	1
Li 2015^[^^59^^]^	Adult	Mannitol vs. HTS**	2014	3	Mod	0	15	0
**Management guided by intracranial pressure**								
Mendelson 2012^[^^60^^]^	Adult	ICP-directed therapy **	2011	0	Mod	1	1	0
Sadaka 2013^[^^61^^]^	Adult	Placement of ICP monitors	1993–2011	0	Low	0	2	0
Su 2014^[^^62^^]^	All	ICP-directed therapy	2013	1	Mod	0	1	0
Yuan 2015^[^^63^^]^	Adult	ICP Monitoring**	2013	1	Mod	0	1	0
*†*Forsyth 2015^[^^64^^]^	All	ICP-directed therapy	2015	1	High	0	1	0
**Cerebrospinal fluid drainage**								
^§^Roberts 1998^[^^32^^]^	All	CSF drainage vs no drainage	1996	0	Low	1	0	0
^§^Meyer 2010^[^^31^^]^	All	CSF drainage	1980–2008	1	Mod	0	0	0
**Posture**								
Fan 2004^[^^65^^]^	All	Therapeutic body positioning**	2003	1	Low	0	0	1
^§^Meyer 2010^[^^31^^]^	All	Adjusting head posture	1980–2008	2	Mod	0	0	0
^§^Meyer 2010^[^^31^^]^	All	Body rotation	1980–2008	0	Mod	0	2	0
**Pressure: other**								
*†Muzevic 2013*^*[*^^*66*^^*]*^	*All*	*The Lund concept*	*2013*	*0*	*High*	*0*	*0*	*0*
**5. Nutrition and glucose management**								
**Nutrition: timing, delivery route and nutritional elements**								
Krakau 2006^[^^67^^]^	Adult	Feeding timing, routes, content**	1993–2003	8	Mod	8	5	1
Perel 2006 ^[^^68^^]^	All	Feeding timing & routes	2006	7	High	2	13	0
^§^Lu 2012^[^^19^^]^	Adult	Early nutritional support	2011	3	Low	2	5	12
Wang 2013^[^^69^^]^	All	Feeding timing, routes, elements**	2012	10	High	0	8	4
Wang 2015^[^^70^^]^	All	Sm. intestine vs gastric feeding**	2013	3	Mod	0	18	1
**Nutrition: Insulin**								
Lei 2012^[^^71^^]^	Adult	Tight vs. conv. glycaemic control	2011	4	Mod	1	0	0
^§^Lu 2012^[^^19^^]^	Adult	Insulin therapy	2011	3	Low	1	1	0
**6. Pharmacological therapies not elsewhere defined**								
**Progesterone**								
^§^Meyer 2010^[^^31^^]^	All	Progesterone	1980–2008	2	Low	5	0	0
^§^Lu 2012^[^^19^^]^	Adult	Progesterone	2011	2	Low	5	0	0
Ma 2012^[^^47^^]^	All	Progesterone vs. placebo	2012	2	High	5	0	0
Wang 2015^[^^72^^]^	All	Progesterone**	1980–2015	5	High	0	0	2
*†Zeng 2015*^*[*^^*73*^^*]*^	*All*	*Progesterone*	*2015*	*6*	*High*	*0*	*1*	*0*
**Bradykinin antagonists**								
^§^Meyer 2010^[^^31^^]^	All	Bradykinin antagonists	1980–2008	3	Low	1	0	0
^§^Lu 2012^[^^19^^]^	Adult	Bradykinin antagonists	2011	1	Low	0	1	2
**Calcium channel blockers**								
Langham 2003^[^^74^^]^	All	Calcium channel blockers	2005	4	Mod	0	0	0
^§^Lu 2012^[^^19^^]^	Adult	Calcium channel blockers	2011	3	Low	0	0	1
**Antifibrinolytic agents**								
Perel 2010^[^^75^^]^	All	Haemostatic agents	2009	2	High	2	0	0
^§^Lu 2012^[^^19^^]^	Adult	Haemostatic agents	2011	1	Low	1	0	2
*†Zehtabchi 2014*^*[*^^*76*^^*]*^	*All*	*Tranexamic acid*	*2014*	*2*	*High*	*0*	*2*	*0*
*†Ker 2015*^*[*^^*77*^^*]*^	*All*	*Antifibrinolytic agents*	*2015*	*2*	*High*	*0*	*2*	*0*
**Monoaminergic agonists**								
Siddall 2005^[^^78^^]^	All	Methylphenidate	2004	0	Low	0	0	0
^§^Meyer 2010[31]	All	Dopamine targeting agents**	1980–2008	0	Mod	0	0	0
*†Forsyth 2006*^*[*^^*79*^^*]*^	*All*	*Monoaminergic agonists*	*2009*	*0*	*High*	*0*	*0*	*0*
Frenette 2012^[^^80^^]^	All	Dopamine agonists	2010	0	Mod	0	0	0
^§^Lu 2012^[^^19^^]^	Adult	Monoaminergic agonists	2011	0	Low	0	0	0
**Aminosteroids**								
*†Roberts 1999*^*[*^^*81*^^*]*^	*All*	*Aminosteroid vs*. *Placebo*	*2006*	*1*	*High*	*0*	*0*	*0*
^§^Lu 2012^[^^19^^]^	Adult	Tirilazad	2011	1	Low	0	0	0
**Pharmacological therapies not elsewhere defined: various (single topic)**								
^§^Meyer 2010^[^^31^^]^	All	Dimethyl sulphoxide	1980–2008	0	Low	0	0	0
^§^Lu 2012^[^^19^^]^	Adult	Pegogortein	2011	1	Low	0	0	0
*†*Alali 2014^[^^82^^]^	Adult	Beta-blockers	2013	1	High	0	0	0
Shen 2015^[^^83^^]^	All	Anticoagulants**	2013	2	Mod	0	0	0
*†Zeiler 2014*^*[*^^*84*^^*]*^	*All*	Tromethamine**	*2014*	*3*	*High*	*0*	*0*	*0*
Sanfilippo 2015^[^^85^^]^	Adult	Neuromuscular blocking agents	2014	3	Low	0	0	0
**7. Glutamate receptor antagonists**								
**Magnesium**								
Arango 2008^[^^86^^]^	All	Magnesium vs. control	2008	1	High	2	0	0
Li 2015^[^^87^^]^	All	Magnesium**	2013	3	Mod	0	0	0
**Glutamate receptor agonists: general**								
Willis 2003^[^^88^^]^	All	EAAI vs. control**	2002	2	High	5	0	0
^§^Meyer 2010^[^^31^^]^	All	Cannabinoids	1980–2008	2	Low	0	5	0
^§^Lu 2012^[^^19^^]^	Adult	EAAI	2011	4	Low	1	0	2
**8. Prehospital and systems of care**								
**Prehospital intubation**								
^§^Lu 2012^[^^19^^]^	Adult	Pre-hospital RSI	2011	1	Low	0	0	0
*†Bossers 2015*^*[*^^*89*^^*]*^	*Adult*	Prehospital intubation**	*2015*	*1*	*High*	*0*	*0*	*0*
**Specialist versus general hospital transfer or care**								
Pickering 2015^[^^90^^]^	All	Prehospital transfer strategies	1998–2012	0	Mod	1	0	0
*†Fuller 2014*^*[*^^*91*^^*]*^	*Adult*	*Specialist neuroscience care*	*2013*	*0*	*High*	*0*	*1*	*0*
**9. Sedation, Pain management, Anaesthesia and Arousal**								
**Sedative agents**								
^§^Meyer 2010^[^^31^^]^	All	Opiods, propofol, midazolam	1980–2008	3	Mod	2	1	6
Roberts 2011^[^^92^^]^	All	Range of sedative agents	2010	10	High	2	0	0
Gu 2014^[^^93^^]^	All	Midazolam vs. propofol	2013	2	Mod	0	10	0
**Ketamine**								
Zeiler 2014^[^^94^^]^	All	Ketamine**	2013	2	High	1	0	0
Wang 2014^[^^95^^]^	All	Ketamine vs opiods**	2014	2	Mod	0	0	1
Cohen 2015^[^^87^^]^	Adult	Ketamine**	2014	3	Mod	0	0	0
**Barbiturates**								
^§^Roberts 1998^[^^32^^]^	All	Barbiturates vs. no barbiturates	1996	2	Low	0	5	2
^§^Meyer 2010^[^^31^^]^	All	Barbiturates	1980–2008	3	Low	2	1	3
^§^Lu 2012^[^^19^^]^	Adult	Barbiturates	2011	2	Low	1	1	5
Roberts 2012^[^^96^^]^	All	Barbiturates	2012	6	High	0	1	2
**Stimulation**								
^§^Meyer 2010^[^^31^^]^	All	Stimulation; sensory, electrical	1980–2008	3	Mod.	1	0	0
*†Wong 2013*^*[*^^*97*^^*]*^	*All*	*Acupuncture*	*2012*	*0*	*High*	*0*	*0*	*0*
**Burst suppression**								
Zeiler 2015^[^^98^^]^	All	Burst suppression**	2015	1	High	0	18	2
**10. Seizure prophylaxis**								
**Anti-epileptic agents**								
Schierhout 1998^[^^99^^]^	All	Anti-epileptic agents	1996	4	Mod	5	3	1
Teasell 2007^[^^100^^]^	All	Any seizure interventions	1980–2005	4	Mod	3	4	2
Zafar 2012^[^^101^^]^	All	Phenytoin vs. levetiracetam	2011	0	High	0	12	1
Thompson 2015^[^^102^^]^	All	Anti-epileptic, neuroprot. agents**	2015	8	High	1	4	0
**11. Steroids**								
**Corticosteroids**								
^§^Roberts 1998^[^^32^^]^	All	Corticosteroids vs. no corticost.	1996	11	Low	2	3	3
Alderson 2005^[^^103^^]^	All	Corticosteroids vs. control	2008	16	High	1	2	0
^§^Meyer 2010^[^^31^^]^	All	Corticosteroids	1980–2008	7	Low	1	5	6
^§^Lu 2012^[^^19^^]^	Adult	Corticosteroids	2011	6	Low	1	0	12
**12. Surgery**								
**Surgery: compared with no surgery and/or with different surgical techniques**								
Sahuquillo 2006^[^^104^^]^	All	Decompressive craniectomy	2008	1	High	4	5	0
^§^Meyer 2010^[^^31^^]^	All	Decompressive craniectomy	1980–2008	2	Mod	7	0	1
Jacob 2011^[^^105^^]^	Paed.	Decompressive craniectomy**	1997–2008	0	Low	0	9	1
Guresir 2012^[^^106^^]^	Paed.	Decompressive craniectomy	2010	0	Low	0	9	1
Bor-Seng-Shu 2012^[^^107^^]^	All	Decompressive craniectomy**	2010	1	Low	1	8	0
^§^Lu 2012^[^^19^^]^	Adult	Decompressive craniectomy	2011	3	Low	6	0	1
Wang 2015^[^^108^^]^	All	Decompressive craniectomy**	2015	3	Mod	2	4	1
**Surgery: timing of surgery (n = 10 RCTs in total)**								
Kim 2014^[^^109^^]^	All	Time to surgery**	1990–2013	2	Low	0	8	0

*Post-dates the systematic review: RCT published subsequent to the systematic review, but would otherwise have met the review inclusion criteria (score of 0 means the review is ‘current’)

^Out of scope: RCT did not meet the systematic review inclusion criteria irrespective of when it was published. RCTs that post-dated a review but would not have met the inclusion criteria were coded to this category.

#True Missing: RCT was published within review search dates and looks to have met the review inclusion criteria (score of 0 means the review is ‘complete’)

†Systematic review was judged to be current, complete and high quality

§Multi-intervention review (included more than one intervention type)

**Included one or more outcomes as inclusion criteria

Abbreviations: Conv. = conventional; Corticost. = corticosteroids; CPP = central perfusion pressure; CSF = cerebrospinal fluid; EAAI = excitatory amino acid inhibitors; Endotrach = endotracheal, HTS = hypertonic saline; ICP = intracranial pressure; Includ. = included, Mod. = moderate; Neuroprot = neuroprotective agents; Normo. = normothermia; Normovent. = normoventilation; Paed. = paediatric; PbtO_2_ = brain tissue oxygen; Pop. = population; PD = post-date the review; Qual. = quality; RBC = red blood cells, RCTs = randomised controlled trials; RSI = rapid sequence intubation; Rx = treatment; S = out of scope; Sm = small, T = true missing, Vs. = versus

#### Currency

**Fig 1:**
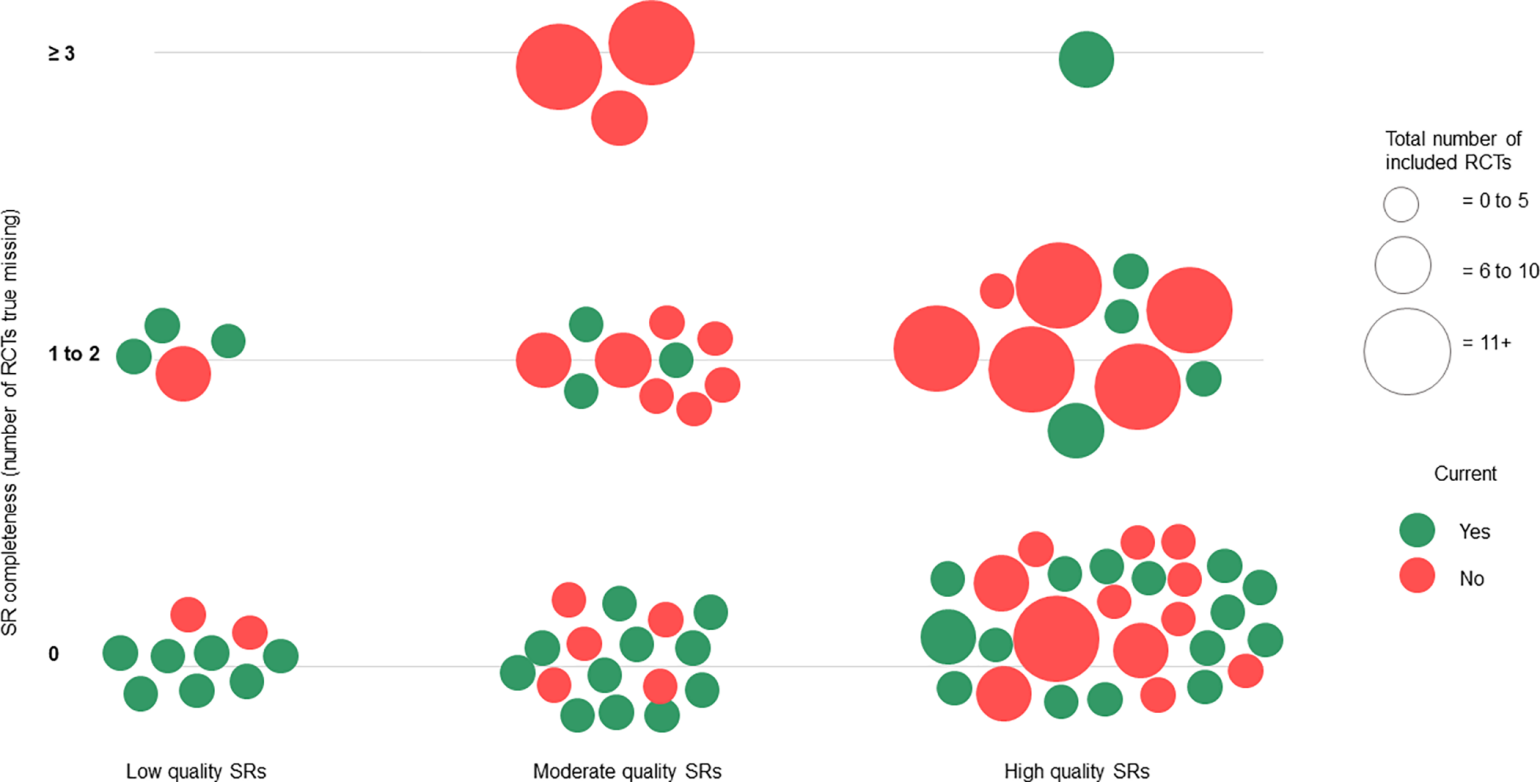
Currency, completeness, and quality of single-intervention systematic reviews. Each bubble represents a single-intervention systematic review (n = 80). The ideal scenario is for bubbles to sit in the bottom right corner (denoting high quality and completeness), and be green in colour (denoting currency). Abbreviations: RCT = randomised controlled trial, SR = systematic review.

Of the 80 single-intervention reviews, approximately half (n = 44, 55.0%) were judged as current, meaning they included the most recently published eligible RCTs (see Table 1; Fig 1). The remainder lacked currency, as there was one RCT (n = 13, 16.3%) two RCTs (n = 8, 10%) or 3 or more RCTs (n = 15, 18.8%) that were published subsequent to the review. When the most recently published systematic review in each intervention area (n = 25) was considered (owing to the inherent disadvantage in assessing currency for older systematic reviews) these numbers improved considerably, with nearly three-quarters of reviews (n = 18, 72.0%) found to be current. The majority (n = 5) of those found to be not current were missing one RCT only. For the five multi-intervention reviews currency was similar, with just under half found to be current (n = 17, 42.5%).

#### Completeness

Of the 80 single-intervention reviews, approximately two-thirds (n = 52, 65.0%) were judged as complete, meaning they included all published RCTs that met their inclusion criteria, relative to when their date of search (see Table 1; Fig 1). The remainder were missing one RCT (n = 16, 20.0%) or two or more RCTs (n = 11, 15.0%) that we judged should have been included. The five multi-intervention reviews fared more poorly on completeness, with half of the 40 individual interventions assessed found to be complete (n = 20, 50%).

#### Quality

Methodological quality of the systematic reviews was variable, with AMSTAR scores ranging from 0 to 10 out of 11 (see S2 Table). Of the 85 systematic reviews included, just under half (n = 38, 44.7%) were rated as high quality, with approximately one-third (n = 31, 36.5%) found to be moderate quality, with the remaining 16 (18.8%) judged as low quality (see Table 1; Fig 1). The five multi-intervention reviews were rated as low [19, 31, 32] or moderate quality [31].

The quality items in which the reviews scored best were the provision of comprehensive search details (n = 74, 87.1%), providing a detailed account of included studies (n = 81, 95.3%), assessing study quality (n = 69, 81.2%) and using appropriate methods for pooling studies (n = 69, 81.2%). Between half to one-third of systematic reviews reported using two independent reviewers (n = 53, 62.4%) or including unpublished studies (n = 45, 52.9%). Similar numbers of systematic reviews were found to have used their quality assessment ratings to interpret review findings (n = 58, 68.2%), or to have explicitly considered publication bias (n = 41, 48.2%). Only one-third of systematic review authors reported a study protocol (n = 29, 34.1%) and provided a full account of included and excluded studies (n = 30, 35.3%). No systematic review included both review-level and included study-level conflict of interest/funding information.

#### Combined currency, completeness and quality of systematic reviews

Across the 80 single-intervention reviews, 16 (20.0%) were judged as meeting all three criteria of being current, complete and high quality (see Table 1). Five of these reviews, on moderate hypothermia [44], the Lund concept [66], monoaminergic agonists [79], specialist neuroscience care [91], and acupuncture [97], did not contain any RCTs. They were either empty reviews, or none of their included studies met our definition of an RCT. No multi-intervention reviews were judged as being current, complete and high-quality.

As such, the following 11 interventions are underpinned by current, complete and high quality systematic review(s) that include one or more RCT: red blood cell transfusion [39], hypothermia [49], management guided by intracranial pressure [64], various pharmacological agents (progesterone [73], antifibrinolytic agents [76, 77], aminosteroids [81], beta-blockers [82], tromethamine [84]), and prehospital intubation [89].

Within the following intervention categories we found no systematic reviews including one or more RCT that are current, complete and high quality: airway, ventilation and oxygenation strategies; nutrition and glucose management; glutamate receptor antagonists; sedation, pain management, anaesthesia and arousal; seizure prophylaxis; corticosteroids; and surgery.

### Randomised controlled trials not included in any systematic reviews

Of the 213 RCTs included, over three-quarters (n = 167, 78.4%) were included in one or more systematic review, leaving 46 RCTs (21.6%) that were not included in any systematic reviews (see Table 2). For approximately two thirds of these RCTs (n = 29, 63.0%), this was because there was no existing systematic review on that intervention topic. The remaining third of these RCTs, (n = 17, 37.0%) post-date the most recently published systematic review in that area, or they were found to be out of the scope of existing systematic reviews.

**Table 2:** Randomised controlled trials not included in any systematic review, with reasons.

Reason	RCT intervention or topic*	RCTs (n =)
**1. Airway, ventilation and oxygenation strategies**		
No SR exists	Early trache.^[^^110^^]^, temperature-corrected (pH-stat) blood gas-guided ventilatory Mx ^[^^111^^]^, normobaric hyperoxia^[^^112^^]^	3
SR exists^^^	Hyperbaric oxygen therapy^[^^113^^]^, Brain tissue oxygen guided Mx^[^^114^^]^	2
**2. Fluid management**		
No SR exists	Fresh frozen plasma^[^^115^^]^	1
SR exists^^^	*Nil*	0
**3. Hypothermia**		
No SR exists	Normothermia (fever control)^[^^116^^]^	1
SR exists^^^	Hypothermia x 6 ^[^^117^^–^^122^^]^	6
**4. Intracranial, cerebral and blood pressure management**		
No SR exists	Vasopressin vs. catecholamines^[^^123^^]^	1
SR exists^^^	CBF- vs. ICP-targeted Mx^[^^124^^]^, hypertonic saline + dextran x 2^[^^125^^,^ ^126^^]^	3
**5. Nutrition and glucose management**		
No SR exists	*Nil*	0
SR exists^^^	Glycaemic control^[^^127^^]^, vit. C^[^^128^^]^, probiotics ^[^^129^^]^, jejunal vs. gastric feed^[^^130^^]^, high protein feed^[^^131^^]^	5
**2. Pharmacological therapies not elsewhere defined**		
No SR exists	Erythropoetin x 3^[^^132^^–^^134^^]^, Cyclosporine x 2^[^^135^^,^ ^136^^]^, Statins x 2 ^[^^137^^,^ ^138^^]^, Prostacyclin^[^^139^^,^ ^140^^]^, Metoclompromide^[^^141^^]^, Cerebrolysin^[^^142^^]^	10
SR exists^^^	Anatibant (different doses) ^[^^143^^]^	1
**3. Glutamate receptor antagonists**		
No SR exists	*Nil*	0
SR exists^^^	*Nil*	0
**4. Prehospital and systems of care**		
No SR exists	Physician prehospital Mx^[^^144^^]^	1
SR exists^^^	Bypass to neurosurg. centre ^[^^145^^]^	1
**5. Sedation, pain management, anaesthesia and arousal**		
No SR exists	*Nil*	0
SR exists^^^	Thiopental vs. propofol^[^^146^^]^, phenobarbitol + phenytoin^[^^147^^]^, auditory stim.^[^^148^^]^	3
**6. Seizure prophylaxis**		
No SR exists	*Nil*	0
SR exists^^^	Lacosamide vs. fosphenytoin^[^^149^^]^	1
**7. Corticosteroids**		
No SR exists	*Nil*	0
SR exists^^^	Hydrocortisone + fludrocortisone^[^^150^^]^, dexamethasone[151]	2
**8. Surgery**		
No SR exists	*Nil*	0
SR exists^^^	Early surgery^[^^152^^]^, decomp. crani. x 2^[^^153^^,^ ^154^^]^, decomp. crani. plus cerebellar incision vs. decomp. crani.^[^^155^^]^, min. invasive surgery ^[^^156^^]^	5

RCTs are grouped together under the 12 broad intervention categories and further classified by whether they meet the inclusion criteria of an existing systematic review, but were omitted for some reason (SR exists) or not (No SR exists).

*Interventions are compared to placebo, control or standard care, unless otherwise stated

^One or more systematic review exists on this topic, but the RCT was published after all systematic review on this topic or was deemed to be out of scope of the existing systematic reviews. Due to the differing inclusion criteria between systematic reviews within a single intervention area, some RCTs were judged as ‘out of scope’ for one systematic review on that topic, whereas they post-dated the publication of another systematic review in the same topic area. Given this, and the fact that the two reasons are both ‘legitimate’ explanations for an RCT to be omitted from a systematic review, we collapsed these two reasons together.

Abbreviations: CBF = Cerebral blood flow, crani. = craniectomy, decomp. = decompressive, FiO_2_ = fraction of inspired oxygen, ICP = Intracranial pressure, Min = minimally, Mx = management, Neurosurg. = neurosurgical, SR = systematic review, Trache. = tracheostomy, vs. = versus

## Discussion

We identified 85 systematic reviews and 213 RCTs in acute management of moderate to severe TBI. The most frequently reviewed interventions were hypothermia, hypertonic saline and/or mannitol and surgery. Approximately half of the systematic reviews lacked currency, in that they did not include most recently published eligible RCT, and one-third of reviews were incomplete, meaning they appeared to miss one or more eligible RCT. When considering only the most recently published systematic review in each intervention, currency increased to approximately 75%. Approximately one-quarter of the RCTs in the acute management of moderate to severe TBI are not included in any systematic review, thus limiting their ability to impact upon practice.

In this study, that less than half of all systematic reviews in acute management of moderate to severe TBI were rated as high quality, with nearly 20% judged as low quality. This is consistent with recent examinations of systematic review quality in biomedical research more broadly [9, 10]. It is therefore not surprising that one-third of systematic reviews referred to a review protocol, and two-thirds lacked transparency around inclusion and exclusion decisions. Additionally, the number of reviews that missed RCTs suggests that searches are not sufficiently comprehensive. It is notable that no review reported both study-level and review-level funding or conflicts of interest. This is particularly problematic given the association between industry funding and favourable results, for both RCTs [157] and systematic reviews [11]. The implication of poor quality systematic reviews is that they may not provide trustworthy evidence to inform clinical practice. While there is meta-epidemiological research showing the correlation between risk of bias in RCTs and overestimation of treatment effects [158], there is limited methodological research into the relationship between systematic review quality and direction or strength of review results.

Despite 85 systematic reviews in this area, the only interventions underpinned by current, complete and high quality evidence are red blood cell transfusion, hypothermia, management guided by intracranial pressure, pharmacological agents (various) and prehospital intubation. Contrasting this is a picture of research waste, with examples of duplication (17 systematic reviews on hypothermia), redundancy (four systematic reviews on hyperventilation with only one RCT ever published) and potentially misleading reviews due to poor quality and/or missing RCTs. The implications for practice recommendations underpinned by such reviews are of concern.

This study is significant in that we have compiled what we believe to be the broadest and most comprehensive record of published, English-language systematic reviews and RCTs in acute management of moderate to severe TBI, highlighting strengths and weakness. Clinicians, decision makers and trialists may use our analysis of a cohort of systematic reviews to inform decision-making, clinical practice guidelines and future research.

We acknowledge a number of limitations. First, to align our work with that of Bragge [14], we excluded non-English and unpublished systematic reviews and RCTs, meaning our evidence map does not encompass these. While we undoubtedly excluded some non-English language RCTs, most of which were in Chinese [14], there are perhaps fewer non-English language systematic reviews, given the propensity for Chinese authors to publish systematic reviews in English [9]. Second, due to resource limitations, all the data extraction, and approximately 20% of the quality assessment, was undertaken by one reviewer, which may have introduced errors [159]. Third, we did not contact systematic review authors directly and therefore may have incorrectly categorised a number of RCTs as missing when in fact they were screened and excluded by the authors. This does, however, reinforce the importance of complete reporting of review methods as recommended by PRISMA [23]. Finally, we did not re-run the searches immediately prior to publication, but given the ‘state-of-the-science’ nature of this work it is unlikely to have influenced the conclusions of the review.

By its nature as an evidence map, this study provides the foundational work for a strategic research agenda [22]. A more nuanced assessment of systematic review and RCT quality [10, 160] and generalisability, explicit consideration of the potential impact of any new (or missing) RCTs on existing review conclusions and consideration of the clinical importance of the question [161] is warranted before new reviews are undertaken. Similarly, with regards to the RCTs on topics not covered by existing systematic reviews, consideration should be given to the clinical relevance and importance of these questions to stakeholders before undertaking new reviews [8].

This study highlights the ongoing challenge for the research community to produce rigorous and comprehensive systematic reviews that incorporate the latest evidence [162, 163]. A number of solutions have been proposed to improve systematic review quality, many of which focus on improved use, training and mandating of reporting checklists, such as PRISMA, by authors, journal editors and peer reviewers [10, 164]. While such approaches have shown promise in improving systematic review quality and completeness of reporting [164], others have called for a more radical change to the way in which secondary research is produced, with closer links between primary and secondary researchers resulting in prospective meta-analyses [9].

One such new approach is Living Systematic Reviews, defined as up to date online summaries of health care research that are updated as new research becomes available [165]. Living Systematic Reviews have been proposed as a way to maintain currency and quality of reviews, while reducing research waste [166, 167]. Living Systematic Reviews are currently being piloted by Cochrane [168] and explored by a number of research teams internationally [169–171]. In TBI, Living Systematic Reviews are being piloted within the Collaborative European NeuroTrauma Effectiveness Research in Traumatic Brain Injury (CENTER-TBI) project [172, 173]. In the CENTER-TBI model, teams of reviewers, with support from methodologists and content experts, are re-running searches every three months and publishing updates in the supplementary material of the published review [12, 174]. Others are making use of larger collaborations [171], citizen science [168], open data platforms [175], and machine learning and other technological enablers [165] to make a ‘living’ evidence model feasible, scalable and sustainable.

For systematic reviews to inform clinical practice, or to influence the primary research agenda, a careful assessment of the nature, strength and credibility of their findings is required [5]. The next logical steps are translation of review findings, if results are conclusive, or more primary research, if review findings are inconclusive [176]. This makes the case for a new piece of work examining the robustness of systematic review conclusions in TBI. We currently have in preparation a formal overview of systematic reviews in acute management of moderate to severe TBI, in which we build on the work presented here [177].

## Conclusion

A substantial number of published systematic reviews of acute management of moderate to severe TBI lack currency, completeness and quality. These shortcomings could affect the robustness of review findings, yielding potentially unreliable evidence underpinning practice recommendations. We highlight both evidence gaps in this area, where consideration could be given to new systematic reviews, and considerable research waste, with much duplicative and redundant effort. Living systematic reviews are being piloted in TBI and offer an opportunity to improve the evidence base informing clinical care and future research in this area.

## Supporting information

S1 File (Search strategies). (DOCX)

S1 Fig (PRISMA flow chart). (DOCX)

S1 Table (Key excluded systematic reviews, with reasons). (DOCX)

S2 Table (Itemised systematic review quality assessment scores). (DOCX)
