# Supplementary material for: The currency, completeness and quality of systematic reviews of acute management of moderate to severe traumatic brain injury: A comprehensive evidence map
Source: PLoS One. 2018 Jun 21;13(6):e0198676. doi: 10.1371/journal.pone.0198676 (PMC6013193; doi:10.1371/journal.pone.0198676)
Supplement: S1 File — (DOCX) [file pone.0198676.s001.docx]

**S1: SEARCH STRATEGIES**

**2016 update search**

Searched Epistemonikos (<http://www.epistemonikos.org/>) on 30 March 2016, using the following search string:

(title:(traumatic brain injury craniocerebral trauma injuries "glasgow coma scale") OR abstract:(traumatic brain injury craniocerebral trauma injuries "glasgow coma scale")) [Filters: protocol=no, min_year=2015, max_year=2016]

**Randomised controlled trial update search**

Searched Cochrane CENTRAL on 30 March 2016, using the following search string:

#1 Cerebrovascular Trauma

#2 BRAIN EDEMA

#3 GLASGOW COMA SCALE

#4 GLASGOW OUTCOME SCALE

#5 UNCONSCIOUSNESS

#6 CRANIOCEREBRAL TRAUMA

#7 ((brain or cerebral or intracranial) adj3 (oedema or edema or swell$)):TI,AB,KY

#8 (Glasgow ADJ3 (coma or outcome) ADJ3 (scale* or score*)):TI,AB,KY

#9 (Unconscious* or coma* or concuss* or ’persistent vegetative state’):TI,AB,KY

#10 ('Rancho Los Amigos Scale'):TI,AB,KY

#11 ((head or crani* or cerebr* or capitis or brain* or forebrain* or skull* or hemisphere* or intra-cran* or inter-cran*) adj3 (injur* or trauma* or damag* or wound* or fracture$ or contusion*)):TI,AB,KY

#12 (Diffuse axonal injury):TI,AB,KY

#13 ((head or crani* or cerebr* or brain* or intra-cran* or inter-cran*) adj3 (haematoma* or hematoma* or haemorrhag* or hemorrhag* or bleed* or pressure)):TI,AB,KY

#14 #1 OR #2 OR #3 OR #4 OR #5 OR #6 OR #7 OR #8 OR #9 OR #10 OR #11 OR #12 OR #13

#15 2015 TO 2016:YR

#16 #14 AND #15
