# Supplementary material for: The currency, completeness and quality of systematic reviews of acute management of moderate to severe traumatic brain injury: A comprehensive evidence map
Source: PLoS One. 2018 Jun 21;13(6):e0198676. doi: 10.1371/journal.pone.0198676 (PMC6013193; doi:10.1371/journal.pone.0198676)
Supplement: S1 Fig — (DOCX) [file pone.0198676.s002.docx]

**S2: PRISMA flow chart**

N = 6 SRs excluded

N = 73 SRs excluded

N = 28 RCTs excluded

N = 194 RCTs included

N = 1,092 SRs screened on title and abstract

N = 672 RCTs screened on title and abstract

N = 47 RCTs screened on full-text

N = 91 SRs screened on full-text

**N = 85 SRs included (combined search yields)**

**N = 213 RCTS included (combined search yields)**

N = 18 SRs included

N = 1,001 SRs excluded

N = 625 RCTs excluded

N = 19 RCTs included

UPDATE SEARCH (MARCH 2016)

MARCH 2015 SEARCH

N = 67 SRs included

N = 73 SRs screened on title and full-text
