# Supplementary material for: The currency, completeness and quality of systematic reviews of acute management of moderate to severe traumatic brain injury: A comprehensive evidence map
Source: PLoS One. 2018 Jun 21;13(6):e0198676. doi: 10.1371/journal.pone.0198676 (PMC6013193; doi:10.1371/journal.pone.0198676)
Supplement: S1 Table — (DOCX) [file pone.0198676.s003.docx]

**S3 TABLE: KEY EXCLUDED SYSTEMATIC REVIEWS**

| **Systematic review** | **Topic** | **Reason for exclusion** |
| --- | --- | --- |
| Han 2014 (1) | Overview of systematic reviews and RCTs of corticosteroids in TBI and SCI | An overview of systematic reviews |
| Costello 2014 (2) | Scoping review of systematic reviews and RCTs of nutrition therapy in TBI | An overview of systematic reviews |
| Batchelor 2012 (3) | Systematic review of the effect of anticoagulation on mortality | Not a systematic review of RCTs (or seeking to include RCTs). Only sought to include observational studies |
| deAmorim 2014 (4) | Combined case report and systematic review of traumatic acute posterior fossa subdural haematoma | Not a systematic review of RCTs (or seeking to include RCTs). Only sought to include case reports |
| Georgiopoulous 2009 (5) | Systematic review of treatments for people in chronic vegetative state or minimally conscious state. | Not acute TBI population (only included studies with people with traumatic injuries that occurred at least 6 months earlier). |
| Kramer 2012 (6) | Hypothermia for people with TBI (described as a critically appraised topic). | Not a systematic review (more of an overview or scoping review, as it includes both primary and secondary research) |
| Galbiati 2014 (7) | Literature review of the effects of open and closed endotracheal suctioning in adults with severe brain injury. | Lack of clear inclusion criteria and included primary and secondary research |
| Maconochie 2010 (8) | Described as a systematic review of interventions to reduce complications of moderate to severe head injury | More of an overview or scoping review, as it includes both primary and secondary research |

**References**

1. Han Z, Lat I, Pollard SR. Safety and efficacy of corticosteroid use in neurologic trauma. Journal of pharmacy practice. 2014;27(5):487-95.

2. Costello LA, Lithander FE, Gruen RL, Williams LT. Nutrition therapy in the optimisation of health outcomes in adult patients with moderate to severe traumatic brain injury: findings from a scoping review. Injury. 2014;45(12):1834-41.

3. Batchelor JS, Grayson A. A meta-analysis to determine the effect of anticoagulation on mortality in patients with blunt head trauma. Br J Neurosurg. 2012;26(4):525-30.

4. de Amorim RL, Stiver SI, Paiva WS, Bor-Seng-Shu E, Sterman-Neto H, de Andrade AF, et al. Treatment of traumatic acute posterior fossa subdural hematoma: report of four cases with systematic review and management algorithm. Acta neurochirurgica. 2014;156(1):199-206.

5. Georgiopoulos M, Katsakiori P, Kefalopoulou Z, Ellul J, Chroni E, Constantoyannis C. Vegetative state and minimally conscious state: a review of the therapeutic interventions. Stereotactic and functional neurosurgery. 2010;88(4):199-207.

6. Kramer C, Freeman WD, Larson JS, Hoffman-Snyder C, Wellik KE, Demaerschalk BM, et al. Therapeutic hypothermia for severe traumatic brain injury: a critically appraised topic. The neurologist. 2012;18(3):173-7.

7. Galbiati G, Paola C. Effects of Open and Closed Endotracheal Suctioning on Intracranial Pressure and Cerebral Perfusion Pressure in Adult Patients With Severe Brain Injury: A Literature Review. J Neurosci Nurs. 2015;47(4):239-46.

8. Maconochie I, Ross M. Head injury (moderate to severe). BMJ clinical evidence. 2010;2010.
