# Supplementary material for: The currency, completeness and quality of systematic reviews of acute management of moderate to severe traumatic brain injury: A comprehensive evidence map
Source: PLoS One. 2018 Jun 21;13(6):e0198676. doi: 10.1371/journal.pone.0198676 (PMC6013193; doi:10.1371/journal.pone.0198676)
Supplement: S2 Table — (DOCX) [file pone.0198676.s004.docx]

**S4 TABLE: ITEMISED SYSTEMATIC REVIEW QUALITY ASSESSMENT SCORES**

| **Systematic review** | **Broad intervention area** | **Protocol** | **Duplicate screen and data extract** | **Search comprehensive** | **Unpublished studies included** | **List of included + excluded studies** | **Included study characteristics** | **Quality assessed** | **Used quality scores in results** | **Appropriate pooling methods** | **Publication bias assessment** | **Study and review conflict of interest** | **Total score/11** | **Quality rating** |
| --- | --- | --- | --- | --- | --- | --- | --- | --- | --- | --- | --- | --- | --- | --- |
| Alali 2014 | Pharmacological therapies | 0 | Y | Y | Y | Y | Y | Y | Y | Y | 0 | 0 | 8 | High |
| Alderson 2005 | Corticosteroids | 0 | Y | Y | Y | 0 | Y | Y | Y | Y | Y | 0 | 8 | High |
| Arango 2008 | Glutamate receptor antagonists | Y | Y | Y | Y | Y | Y | Y | Y | Y | 0 | 0 | 9 | High |
| Banks 2008 | Intracranial, cerebral and BP Mx | 0 | 0 | Y | 0 | 0 | 0 | Y | Y | 0 | 0 | 0 | 3 | Low |
| Bennett 2012 | Airway, ventilation and oxygenation | Y | Y | Y | Y | Y | Y | Y | Y | Y | 0 | 0 | 9 | High |
| Bor-SengShu 2012 | Surgery | 0 | 0 | 0 | 0 | Y | Y | 0 | 0 | Y | 0 | 0 | 3 | Low |
| Bossers 2015 | Prehospital and systems of care | 0 | Y | Y | Y | 0 | Y | Y | Y | Y | Y | 0 | 8 | High |
| Boutin 2015 | Fluid management | Y | Y | Y | 0 | 0 | Y | Y | Y | Y | Y | 0 | 8 | High |
| Cohen 2015 | Sedation, pain Mx, anaesthesia | 0 | Y | Y | Y | 0 | Y | Y | 0 | Y | Y | 0 | 7 | Mod |
| Crossley 2014 | Hypothermia | Y | Y | Y | Y | Y | Y | Y | Y | Y | Y | 0 | 10 | High |
| Fan 2004 | Intracranial, cerebral and BP Mx | 0 | 0 | Y | Y | 0 | Y | 0 | 0 | 0 | 0 | 0 | 3 | Low |
| Forsyth 2006 | Pharmacological therapies | Y | Y | Y | Y | Y | Y | Y | Y | Y | 0 | 0 | 9 | High |
| Forsyth 2015 | Intracranial, cerebral and BP Mx | Y | Y | Y | Y | Y | Y | Y | 0 | Y | Y | 0 | 9 | High |
| Fox 2010 | Hypothermia | Y | Y | Y | 0 | 0 | Y | Y | Y | Y | Y | 0 | 8 | High |
| Frenette 2012 | Pharmacological therapies | 0 | Y | Y | 0 | 0 | Y | Y | Y | Y | 0 | 0 | 6 | Mod |
| Fuller 2014 | Prehospital and systems of care | Y | 0 | Y | Y | Y | Y | Y | Y | Y | Y | 0 | 9 | High |
| Georgiou 2013 | Hypothermia | Y | 0 | Y | Y | 0 | Y | Y | Y | Y | Y | 0 | 8 | High |
| Gu 2014 | Sedation, pain Mx, anaesthesia | 0 | 0 | 0 | 0 | 0 | Y | 0 | 0 | Y | Y | 0 | 3 | Low |
| Guresir 2012 | Surgery | 0 | 0 | 0 | 0 | 0 | Y | 0 | 0 | 0 | 0 | 0 | 1 | Low |
| Harris 2002 | Hypothermia | 0 | Y | Y | 0 | 0 | Y | Y | Y | Y | Y | 0 | 7 | Mod |
| Harris 2012 | Hypothermia | Y | 0 | Y | Y | Y | Y | Y | Y | Y | 0 | 0 | 8 | High |
| Henderson 2003 | Hypothermia | 0 | 0 | Y | Y | Y | Y | 0 | 0 | Y | 0 | 0 | 5 | Mod |
| Jacob 2011 | Surgery | 0 | 0 | 0 | 0 | 0 | 0 | 0 | 0 | 0 | 0 | 0 | 0 | Low |
| Kamel 2011 | Intracranial, cerebral and BP Mx | 0 | Y | Y | Y | 0 | Y | Y | 0 | Y | Y | 0 | 7 | Mod |
| Ker 2015 | Pharmacological therapies | Y | Y | Y | Y | Y | Y | Y | Y | Y | Y | 0 | 10 | High |
| Kim 2014 | Surgery | 0 | 0 | Y | 0 | 0 | Y | Y | 0 | 0 | 0 | 0 | 3 | Low |
| Krakau 2006 | Nutrition and glucose management | 0 | Y | Y | Y | 0 | 0 | Y | 0 | Y | 0 | 0 | 5 | Mod |
| Langham 2003 | Pharmacological therapies | Y | 0 | Y | Y | 0 | Y | Y | Y | Y | 0 | 0 | 7 | Mod |
| Lazaridis 2014 | Airway, ventilation and oxygenation | 0 | 0 | Y | 0 | 0 | Y | Y | Y | Y | 0 | 0 | 5 | Mod |
| Lei 2012 | Nutrition and glucose management | 0 | Y | Y | 0 | 0 | Y | Y | Y | Y | Y | 0 | 7 | Mod |
| Li 2014 | Hypothermia | 0 | 0 | Y | 0 | 0 | Y | 0 | 0 | Y | Y | 0 | 4 | Mod |
| Li 2015 | Intracranial, cerebral and BP Mx | 0 | Y | 0 | 0 | 0 | Y | Y | 0 | Y | Y | 0 | 5 | Mod |
| Li 2015 | Glutamate receptor antagonists | 0 | Y | Y | 0 | 0 | Y | Y | Y | Y | Y | 0 | 7 | Mod |
| Lourens 2014 | Intracranial, cerebral and BP Mx | 0 | Y | Y | Y | 0 | Y | Y | Y | Y | Y | 0 | 8 | High |
| Lu 2012 | Multi-intervention review | 0 | 0 | Y | Y | 0 | Y | 0 | 0 | 0 | 0 | 0 | 3 | Low |
| Ma 2012 | Pharmacological therapies | Y | Y | Y | Y | Y | Y | Y | Y | Y | Y | 0 | 10 | High |
| Ma 2013 | Hypothermia | 0 | Y | 0 | 0 | 0 | Y | Y | Y | Y | Y | 0 | 6 | Mod |
| Madden 2015 | Hypothermia | 0 | 0 | 0 | 0 | 0 | Y | Y | 0 | Y | 0 | 0 | 3 | Low |
| McDonough 2004 | Airway, ventilation and oxygenation | 0 | Y | Y | Y | 0 | Y | Y | Y | 0 | 0 | 0 | 6 | Mod |
| McIntyre 2003 | Hypothermia | 0 | Y | Y | Y | 0 | Y | Y | Y | Y | Y | 0 | 8 | High |
| Mendelson 2012 | Intracranial, cerebral and BP Mx | 0 | Y | Y | Y | 0 | Y | Y | 0 | Y | 0 | 0 | 6 | Mod |
| Meyer 2010 | Multi-intervention review | 0 | 0 | Y | 0 | 0 | Y | Y | 0 | 0 | 0 | 0 | 3 | Low |
| Meyer 2010 | Multi-intervention review | 0 | 0 | Y | 0 | 0 | Y | Y | Y | 0 | 0 | 0 | 4 | Mod |
| Meyer 2010 | Multi-intervention review | 0 | 0 | Y | 0 | 0 | Y | Y | Y | 0 | 0 | 0 | 4 | Mod |
| Muzevic 2013 | Intracranial, cerebral and BP Mx | Y | Y | Y | Y | Y | Y | Y | Y | Y | 0 | 0 | 9 | High |
| Nangunoori 2012 | Airway, ventilation and oxygenation | 0 | 0 | Y | 0 | 0 | Y | 0 | 0 | Y | 0 | 0 | 3 | Low |
| Nishijima 2012 | Fluid management | 0 | Y | Y | 0 | Y | Y | Y | Y | Y | 0 | 0 | 7 | Mod |
| Perel 2006 | Nutrition and glucose management | Y | 0 | Y | 0 | Y | Y | Y | Y | Y | Y | 0 | 8 | High |
| Perel 2010 | Pharmacological therapies | Y | Y | Y | Y | Y | Y | Y | Y | Y | Y | 0 | 10 | High |
| Peterson 2008 | Hypothermia | 0 | Y | Y | 0 | 0 | Y | Y | Y | Y | 0 | 0 | 6 | Mod |
| Pickering 2015 | Prehospital and systems of care | 0 | 0 | Y | Y | Y | Y | 0 | 0 | Y | 0 | 0 | 5 | Mod |
| Rickard 2014 | Intracranial, cerebral and BP Mx | 0 | 0 | 0 | 0 | 0 | 0 | Y | Y | Y | Y | 0 | 4 | Mod |
| Roberts 1997 | Airway, ventilation and oxygenation | Y | Y | Y | 0 | Y | Y | Y | Y | 0 | 0 | 0 | 7 | Mod |
| Roberts 1998 | Multi-intervention review | 0 | 0 | Y | Y | 0 | Y | 0 | 0 | 0 | 0 | 0 | 3 | Low |
| Roberts 1999 | Pharmacological therapies | Y | 0 | Y | Y | Y | Y | Y | Y | Y | Y | 0 | 9 | High |
| Roberts 2011 | Sedation, pain Mx, anaesthesia | Y | Y | Y | Y | 0 | Y | Y | Y | Y | 0 | 0 | 8 | High |
| Roberts 2012 | Sedation, pain Mx, anaesthesia | Y | Y | Y | Y | Y | Y | Y | 0 | Y | Y | 0 | 9 | High |
| Sadaka 2012 | Hypothermia | 0 | 0 | Y | 0 | 0 | Y | 0 | 0 | 0 | 0 | 0 | 2 | Low |
| Sadaka 2013 | Intracranial, cerebral and BP Mx | 0 | 0 | 0 | 0 | 0 | Y | 0 | 0 | 0 | 0 | 0 | 1 | Low |
| Sahuquillo 2006 | Surgery | Y | 0 | Y | Y | Y | Y | Y | Y | Y | 0 | 0 | 8 | High |
| Sanfilippo 2015 | Pharmacological therapies | 0 | 0 | Y | 0 | 0 | Y | 0 | 0 | Y | 0 | 0 | 3 | Low |
| Saxena 2008 | Hypothermia | Y | Y | Y | Y | Y | Y | Y | Y | Y | Y | 0 | 10 | High |
| Schierhout 1998 | Seizure | 0 | 0 | Y | Y | 0 | Y | Y | Y | Y | 0 | 0 | 6 | Mod |
| Shen 2015 | Pharmacological therapies | 0 | Y | Y | 0 | Y | Y | Y | Y | Y | 0 | 0 | 7 | Mod |
| Siddall 2005 | Pharmacological therapies | 0 | 0 | 0 | 0 | 0 | Y | 0 | 0 | 0 | 0 | 0 | 1 | Low |
| Su 2014 | Intracranial, cerebral and BP Mx | 0 | Y | Y | 0 | 0 | Y | Y | 0 | Y | Y | 0 | 6 | Mod |
| Sydenham 2009 | Hypothermia | Y | 0 | Y | Y | Y | Y | Y | Y | Y | 0 | 0 | 8 | High |
| Teasell 2007 | Seizure | 0 | Y | Y | 0 | 0 | Y | Y | Y | 0 | 0 | 0 | 5 | Mod |
| Thompson 2015 | Seizure | Y | Y | Y | Y | Y | Y | Y | Y | Y | Y | 0 | 10 | High |
| Wakai 2013 | Intracranial, cerebral and BP Mx | Y | Y | Y | Y | Y | Y | Y | Y | Y | 0 | 0 | 9 | High |
| Wang 2013 | Nutrition and glucose management | 0 | Y | Y | 0 | Y | Y | Y | Y | Y | Y | 0 | 8 | High |
| Wang 2014 | Sedation, pain Mx, anaesthesia | 0 | Y | Y | 0 | Y | Y | Y | Y | Y | 0 | 0 | 7 | Mod |
| Wang 2015 | Surgery | 0 | Y | Y | 0 | 0 | Y | 0 | 0 | Y | Y | 0 | 5 | Mod |
| Wang 2015 | Nutrition and glucose management | 0 | Y | Y | 0 | 0 | Y | Y | Y | Y | Y | 0 | 7 | Mod |
| Wang 2015 | Pharmacological therapies | Y | Y | Y | 0 | 0 | Y | Y | Y | Y | Y | 0 | 8 | High |
| Willis 2003 | Glutamate receptor antagonists | Y | Y | Y | Y | Y | Y | Y | Y | Y | 0 | 0 | 9 | High |
| Wong 2013 | Sedation, pain Mx, anaesthesia | Y | Y | Y | Y | Y | Y | Y | Y | Y | Y | 0 | 10 | High |
| Yuan 2015 | Intracranial, cerebral and BP Mx | 0 | Y | 0 | 0 | 0 | Y | Y | Y | Y | Y | 0 | 6 | Mod |
| Zafar 2012 | Seizure | 0 | Y | Y | Y | 0 | Y | Y | Y | Y | Y | 0 | 8 | High |
| Zehtabchi 2014 | Pharmacological therapies | Y | Y | Y | Y | 0 | Y | Y | Y | Y | 0 | 0 | 8 | High |
| Zeiler 2014 | Pharmacological therapies | 0 | Y | Y | Y | 0 | Y | Y | Y | Y | Y | 0 | 8 | High |
| Zeiler 2014 | Sedation, pain Mx, anaesthesia | 0 | Y | Y | Y | 0 | Y | Y | Y | Y | Y | 0 | 8 | High |
| Zeiler 2015 | Pharmacological therapies | 0 | Y | Y | Y | 0 | Y | Y | Y | Y | Y | 0 | 8 | High |
| Zeng 2015 | Pharmacological therapies | 0 | Y | Y | Y | 0 | Y | Y | Y | Y | Y | 0 | 8 | High |
| Zhang 2015 | Hypothermia | 0 | Y | Y | 0 | 0 | Y | Y | Y | Y | Y | 0 | 7 | Mod |
| **Number of systematic reviews that scored ‘Yes’ for each item (maximum possible score = 85)** | | 29 | 53 | 74 | 45 | 30 | 81 | 69 | 58 | 69 | 41 | 0 |  |  |
| **Percentage of systematic reviews that scored ‘Yes” for each item** | | 34.1% | 62.4% | 87.1% | 52.9% | 35.3% | 95.3% | 81.2% | 68.2% | 81.2% | 48.2% | 0.0% |  |  |

**Table legend**

The full AMSTAR checklist questions for a each domain are as follows:

1. Protocol: Was an 'a priori' design provided?
2. Duplicate screen and data extract: Was there duplicate study selection and data extraction?
3. Search comprehensive: Was a comprehensive literature search performed?
4. Unpublished studies included: Was the status of publication (i.e. grey literature) used as an inclusion criterion?
5. List of included and excluded studies: Was a list of studies (included and excluded) provided?
6. Included study characteristics: Were the characteristics of the included studies provided?
7. Quality assessed: Was the scientific quality of the included studies assessed and documented?
8. Used quality scores in results: Was the scientific quality of the included studies used appropriately in formulating conclusions?
9. Appropriate pooling methods: Were the methods used to combine the findings of studies appropriate?
10. Publication bias assessment: Was the likelihood of publication bias assessed?
11. Study and review conflict of interest: Was the conflict of interest included?

Abbreviations: BP = Blood pressure, Mx = Management
